# Supplementary material for: Pyrogallol-Phloroglucinol-6,6-Bieckol Alleviates Obesity and Systemic Inflammation in a Mouse Model by Reducing Expression of RAGE and RAGE Ligands
Source: Mar Drugs. 2019 Oct 28;17(11):612. doi: 10.3390/md17110612 (PMC6891643; doi:10.3390/md17110612)
Supplement: Supplementary file 1 [file marinedrugs-17-00612-s001.pdf]

Table S1. List of antibodies for Immunoassay

Antibody name	Company	Catalogue number	Dilution rate	Application
CD86	Santa cruz Biotechnology	sc-19617	1:100	Immunohistochemistry
CD163	Santa cruz Biotechnology	sc-20066	1:100	Immunohistochemistry
RAGE	Santa cruz Biotechnology	sc-365154	1:100	Immunohistochemistry
			1:200	ELISA
AGEs	Abcam	ab 23722	1:1000	ELISA
HMGB1	Abcam	ab 18256	1:1000	ELISA
S100β	Abcam	ab 52642	1:500	ELISA
β-actin	Cell signaling	4967S	1:1000	Immunoblotting

Table S2. List of primer for quantitative polymerase chain reaction

Gene		Primer
CD86	Forward	5'-AGCAACTAATGCTGAAAGCACA-3′
	Reverse	5'-CTCAGACCTGCCAAAGTTCTCT-3′
CD80	Forward	5'-AGCAACTAATGCTGAAAGCACA-3′
	Reverse	5'-CTCAGACCTGCCAAAGTTCTCT-3′
CD163	Forward	5'- AGTGCCTCCCAAAAATGACTT -3′
	Reverse	5'- GACCAATAGAATGGCTCCACA-3′
CD206	Forward	5'-AGCAACTAATGCTGAAAGCACA-3′
	Reverse	5'-CTCAGACCTGCCAAAGTTCTCT-3′
TNF-α	Forward	5'-TTCTGTCTACTGAACTTCGGGGTGATCGGTCC-3′
	Reverse	5'-GTATGAGATAGCAAATCGGCTGACGGTGTGGG-3′
IL-1β	Forward	5'-CCTTTTCGTGAATGAGCAGAC -3′
	Reverse	5'-AGAATGTGCCATGGTTTCTTG-3′
β-actin	Forward	5'-ACAAAGCTGTTCAGTGTCTCCA-3′
	Reverse	5'-CTCCGTTTCCAGAATACACACA-3′

Figure S1. Inhibiting effects of PPB on the expression of RAGE in visceral fat

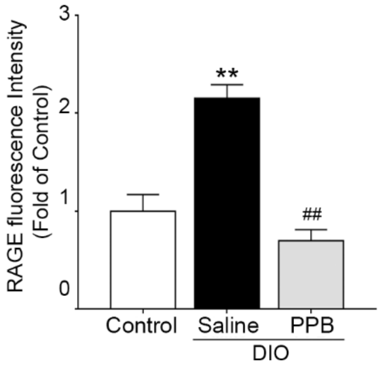

Fluorescence intensity of RAGE in visceral fat. Significance represented as **, $p < 0.01$ versus Control; ##, $p < 0.01$ versus DIO/Saline. DIO, diet-induced obesity; PPB, pyrogallol-phloroglucinol-6,6-bieckol; RAGE, receptor for advanced glycation end-products.

Figure S2. Regulating effects of PPB on M1 and M2 type macrophage differentiation in visceral fat

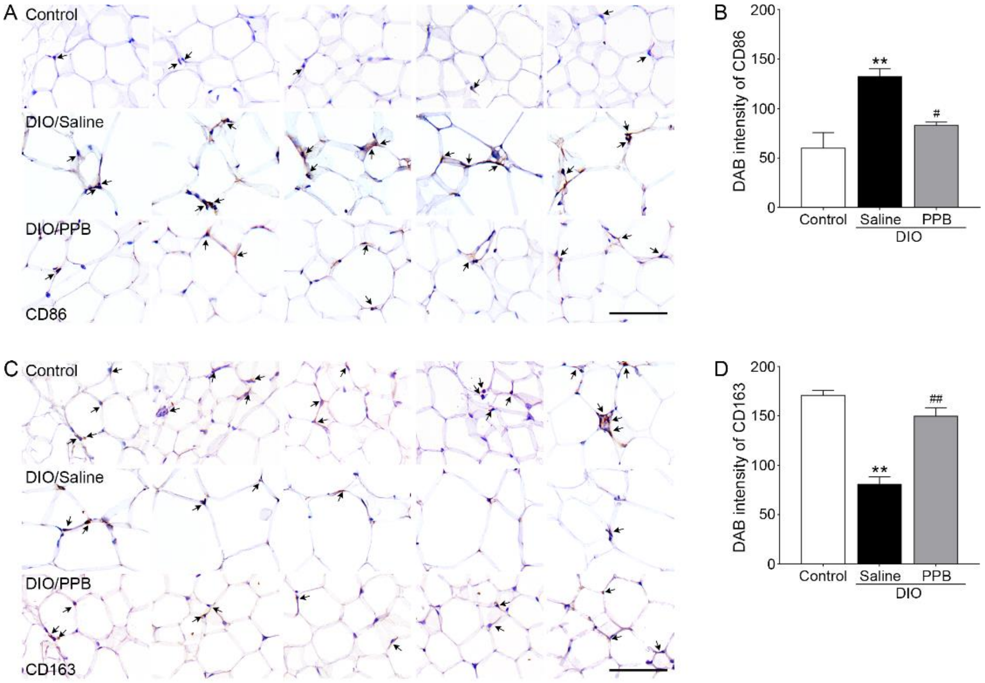

(A) Immunohistochemical images (CD86) and (B) quantified intensity graph are measured in visceral fat. (C) Immunohistochemical images (CD163) and (D) quantified intensity graph are measured in visceral fat. Scale bar = 200 μm; x400 magnification. The significance represented as **, p <0.01 versus Control; #, p <0.05 and ##, p <0.01 versus DIO/Saline. DAB, 3, 3-diaminobenzidine; DIO, diet-induced obesity; PPB, pyrogallol-phloroglucinol-6,6-bieckol

Figure S3. Visceral fat protein purification for control, DIO/Saline and PPB

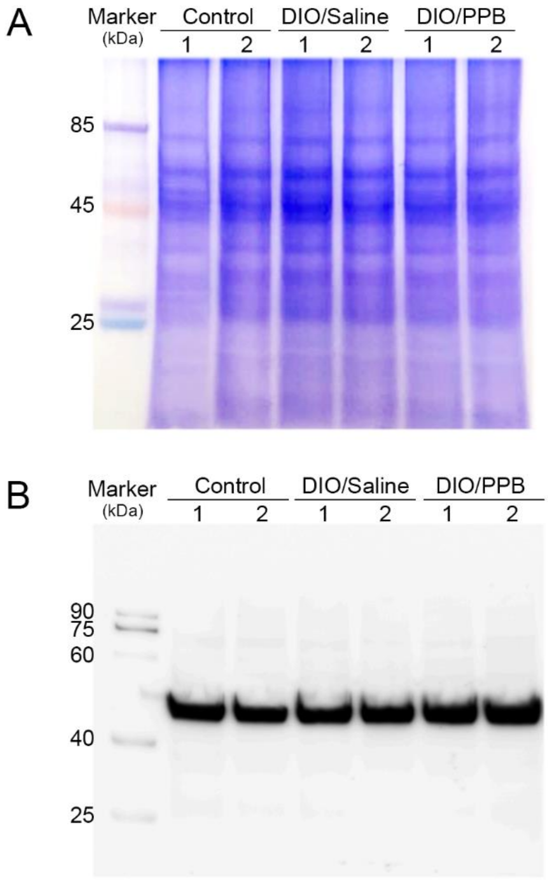

(A) Coomassie blue staining and (B) immunoblotting for β-actin were used for visceral fat protein purification. DIO, diet-induced obesity; PPB, pyrogallol-phloroglucinol-6,6-bieckol
